# What and How Can Physical Activity Prevention Function on Parkinson's Disease?

**DOI:** 10.1155/2020/4293071

**Published:** 2020-02-13

**Authors:** Baozhu Fan, Riffat Jabeen, Bing Bo, Chunlei Guo, Mengjie Han, Hui Zhang, Juan Cen, Xinying Ji, Jianshe Wei

**Affiliations:** ^1^Laboratory of Brain Function and Disease, Institute for Brain Sciences Research, School of Life Sciences, Henan University, China; ^2^School of Physical Education, Henan University, Kaifeng, 475004 Henan, China; ^3^Henan International Joint Laboratory of Nuclear Protein Regulation, Kaifeng 475004, China

## Abstract

**Aim:**

This study was aimed at investigating the effects and molecular mechanisms of physical activity intervention on Parkinson's disease (PD) and providing theoretical guidance for the prevention and treatment of PD.

**Methods:**

Four electronic databases up to December 2019 were searched (PubMed, Springer, Elsevier, and Wiley database), 176 articles were selected. Literature data were analyzed by the logic analysis method.

**Results:**

(1) Risk factors of PD include dairy products, pesticides, traumatic brain injury, and obesity. Protective factors include alcohol, tobacco, coffee, black tea, and physical activity. (2) Physical activity can reduce the risk and improve symptoms of PD and the beneficial forms of physical activity, including running, dancing, traditional Chinese martial arts, yoga, and weight training. (3) Different forms of physical activity alleviate the symptoms of PD through different mechanisms, including reducing the accumulation of *α*-syn protein, inflammation, and oxidative stress, while enhancing BDNF activity, nerve regeneration, and mitochondrial function.

**Conclusion:**

Physical activity has a positive impact on the prevention and treatment of PD. Illustrating the molecular mechanism of physical activity-induced protective effect on PD is an urgent need for improving the efficacy of PD therapy regimens in the future.

## 1. Introduction

Parkinson's disease (PD) is a second common neurodegenerative disease all over the world [1]. Most of the patients are between 50 and 60 years old. As the aging of population increases, the risk of Parkinson's disease increases accordingly, and the incidence in young- and middle-aged people increases. As a chronic disease, PD has a long course and is prone to recurrence. The reduction of dopamine (DA) is the leading cause of PD in previous studies [2]. The consequent loss of the neurotransmitter DA in the striatum leads to the primary motor symptoms of PD, namely, bradykinesia, tremor, rigidity, and postural instability [3–5]. At present, the clinical trials are valid only for symptom management; no medications have proved effective in stopping the disease process [4]. Therefore, revealing the pathological mechanism of PD is extremely important for the effective prevention and treatment of this disease.

The character of brain tissue from PD patients is the degeneration of dopaminergic neurons in the substantia nigra pars compacta of the midbrain, with the concomitant loss of their axons which project to the striatum along the nigrostriatal pathway. Along with the neuronal loss, the appearance of insoluble cytoplasmic inclusions (Lewy bodies, LB) and insoluble fibrils (Lewy neurites, LN) is also a neuropathological hallmark of PD. The main composition of the LB and LN is the *α*-synuclein (*α*-syn) protein [6]. PD can usually be divided into familial and sporadic [7]. Clinical statistics show that the number of familial patients accounts for about 10% of all patients. The leading cause of familial patients is gene mutation, such as PARKIN, DJ-1, PINK1, and ATP13A2 [8], while the main causes of sporadic patients are associated with oxidative stress, neuroinflammation, mitochondrial dysfunction, and environmental factors, such as drugs and pesticides [9].

## 2. Epidemiology Study of Parkinson's Disease

### 2.1. Incidence of Parkinson's Disease

A study of data from 2005 to 2018 indicated that PD is one of the most common neurodegenerative diseases worldwide (second only to Alzheimer's disease). The influence factors in the study of PD were mainly divided into prospective studies and case-control studies. In the world's high-income countries, the median incidence of PD is 14/100,000, and the rate is 160/100,000 in people aged 65 or older [10]. Among the 40-year-old American population, the risk of PD in men is about 2%, while in women is 1.3% [11]. The age-adjusted prevalence of PD reflects morbidity and mortality, which in Africa is lower than that in Europe, the United States, and Asia [12–14]. Currently, there are fewer morbidity data related to racial or ethnic, but the incidence varies according to the current research. A study from a large medical institution in the United States indicated that the incidence in Blacks is higher than that in Whites. The age-adjusted and gender-adjusted incidence of PD was highest among Hispanics (16.6/100,000), followed by non-Hispanic whites (13.6/100,000), Asian (11.3/100,000), and Blacks (10.2/100,000). Another study based on beneficiaries of US health insurance suggested that the incidence of PD in Whites was also higher than that in Blacks or Asians [15–17]. The incidence of PD increases with age and reaches a maximum at 80 years old. In the Chinese population aged 60 years, the incidence of PD is more than 1%.

### 2.2. Risk Factors and Protective Factors of Parkinson's Disease

The risk factors of pathogenicity and morbidity in PD are various. We have found that dairy consumption is positively correlated with the incidence of PD through a series of studies related to aging, cancer prevention, and nutrition [18–20]. The risk of PD in the study sample from the Honolulu-Asian Ageing Study (HAAS) and Cancer Prevention and II Nutritional Research (CPS-IIN) increased with the extension of plantation time [21, 22]. This result is consistent with agricultural health research, which indicates that exposure to pesticides increases the risk of PD. Pesticides could cause oxidative stress and disturb mitochondrial function [23]. Traumatic brain injury can lead to disruption of the blood-brain barrier, impaired mitochondrial function, and accumulation of brain *α*-syn protein, all of these may lead to an increased risk of PD after exposure to such injury [24]. A cohort study in Finland found that overweight (i.e., BMI 27-29.9) or obesity (i.e., BMI ≥ 30) will bring a high-risk factor for PD [25, 26].

**Figure 1:**
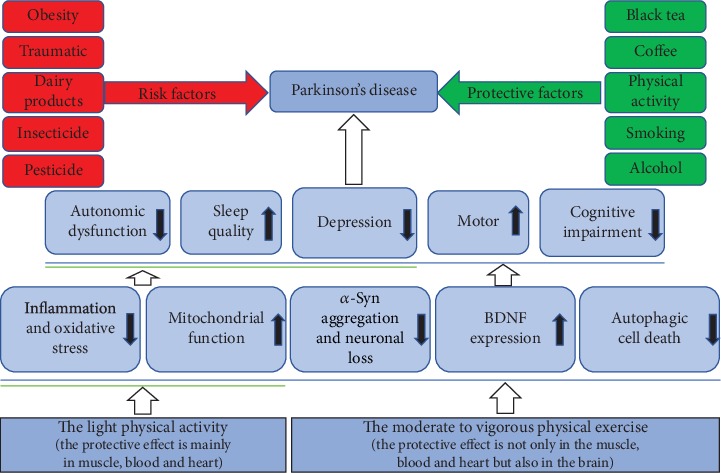
A schematic of physical activity on Parkinson's disease. Risk factors for Parkinson's disease include dairy intake, prolonged exposure to pesticides, traumatic brain injury, and obesity. The protective factors on Parkinson's disease include alcohol intake, tobacco smoking, coffee, black tea, and physical activity. Common forms of physical activity that benefit Parkinson's disease include running, dancing, traditional Chinese martial arts, yoga, and weight training. Physical exercise can reduce *α*-Syn protein aggregation, alleviate neuronal death, regulate inflammation and oxidative stress, promote BDNF activity, modulate neuronal autophagy, and enhance mitochondrial function. Physical activity can improve motor capacity including strength, balance, and flexibility and also enhance the nonmotor symptoms, alleviate cognitive impairment, and improve depression.

The protective factors of PD from numerous studies are also diverse. Compared with nondrinkers, longitudinal studies support a viewpoint that drinkers had a slight decreased risk of PD, which was consistent with the effect of alcoholic beverages on urate levels in the body [27, 28]. Coffee drinkers have a lower risk of PD than other people who do not drink coffee, which has been confirmed in several prospective investigations [29, 30]. The ingredients of black tea may help reduce the risk of PD, but green tea does not have the same effect [31] [32]. With the extension of smoking time, the risk of PD is reduced by up to 70%, and it has increased with time since the quitters quit smoking [33–35]. Besides, the use of antihypertensive drugs [36, 37], physical activity, and a healthy diet [38] can effectively reduce the risk and relieve the symptoms of PD patients (Figure 1).

## 3. Physical Activity Prevention and Parkinson's Disease

### 3.1. Physical Activity Can Reduce the Risks of Parkinson's Disease

Physical activity plays an active role in the prevention and treatment of PD. The relationship between physical activity and risk of PD was first reported in the Nurses' Health Study and HPFS, and later in five other longitudinal studies (Harvard Alumni Health Study, CPS-IIN, NIH-AARP Diet and Health Research, Finnish Mobile Clinic Research, and the Swedish National March Cohort Study). The results of prospective epidemiological studies suggest that active physical activity reduces the risk of PD in men, but the mechanism is uncertain [39–42]. There is no strong supporting evidence for the hypothesis that physical activity can prevent male PD in the Harvard Alumni Health Study. Nevertheless, a smaller sample size study shows a negative and nonsignificant association between physical activity and PD [43]. A study of 143,325 participants from CPS-II-N has found that vigorous activity was associated with PD in men and women, while a reduction in PD risk through moderate to vigorous activity [44]. The study of 213,701 participants of NIH-AARP Diet and Health Study cohort also confirmed this view that higher levels of moderate to vigorous activities at ages 35-39 or in the past ten years as reported in 1996-1997 were associated with low PD incidence after 2000, which was with a significant dose-response relationship. Compared to individuals who were inactive during the two periods, the risk of PD reduced by approximately 40% in the further analysis [45]. Another study from the Swedish National March cohort showed that the total amount of daily activity was associated with a lower risk of PD, but women's correlation was not apparent to men [46].

### 3.2. Multiple Physical Activities Can Improve Motor and Nonmotor Symptoms of Parkinson's Disease

Mehrholz et al. and Herman et al.'s study found that running can improve gait and physical fitness and better safety [47, 48]. Aguiar et al. indicated that regular dancing has the benefit of balance and mobility in PD patients [49]. The rhythmic music used in dancing could activate neurons that are conducive to motor control, accompanied by increasing blood flow in the hippocampus, frontal, temporal, and parietal cortex. This promotion of neural plasticity improves movement, balance, and cognition of the body [50]. Also, Tai Chi and Qigong could upgrade the motor function and balance ability of patients with mild to moderate PD [51–53]. Yoga provides modest improvements in motor functions, mobility, balance, flexibility, and strength in upper and lower limbs, while helps reduce the fear of falls in Parkinson's patients [54].

Physical activity treatment could also improve nonmotor functions in PD patients. In the meantime, physical activity could regulate autonomic dysfunction, including improvement of cardiac sympathetic regulation in PD patients [55], but the effects on other systems are unclear [56]. Resistance training, Qigong, and other types of physical activity could improve sleep quality in PD patients [57] [58]. Another benefit of physical activity to PD patients is cognitive impairment alleviation. David et al. found that 24 months of progressive resistance training may improve attention and working memory in nondemented patients with mild to moderate PD patients [59]. McKee and Hackney indicated that tango participants improved on disease severity, spatial cognition, balance, and executive function [60]. Consistent with the above studies, Cruise et al. found that 12 weeks of progressive aerobic and anabolic physical activity had selective benefits for cognitive functioning by improving frontal lobe-based executive function [61]. As a safe, broad-spectrum intervention, physical activity could also have positive effects on mood, cognition, and sleep for PD patients. Therefore, physical activity could enhance the chances of recovery through improvement in the mood and the nervous system in elderly PD patients [62]. Based on the Feldenkrais method, Teixeira-Machado et al. indicated that 50 sessions of physical therapy programs could promote cognitive function for ages between 50 and 70 PD patients [63]. More and more evidences from numerous studies suggested that physical activity could enhance motor and nonmotor symptoms of PD patients, but the mechanism of exercise in relieving PD symptoms needs further study.

### 3.3. Therapy Regimen by Comparing the Effects of Different Intensities of Physical Activity on PD

The light physical activity (VO_2Max_ 40%–50%) reduces tumor necrosis factor alpha (TNF-alpha) in the skeletal muscle [64] and the thiobarbituric acid reactive substances (TBARS) in the soleus muscles [65]. Linke's study found that physical activity can not only reduce the expression of inflammatory cytokines in the blood but also enhance the activity of free radical scavengers [66]. Schulze's findings suggest that physical activity enhances mitochondrial biogenesis in the vascular endothelium through a shear stress-dependent mechanism [67]. Vettor's findings demonstrate that physical activity promotes endothelial NO synthase- (eNOS-) dependent mitochondrial biogenesis in the heart, which behaves as an essential step in cardiac glucose transport [68]. The light activity can reduce oxidative stress and enhance mitochondrial biogenesis in skeletal muscle, blood, and heart. These protective effects can improve autonomic dysfunction [55], sleep quality [57], and depression [57] in PD patients.

The moderate to vigorous physical activity (VO_2Max_ 50%–80%), 40-60 mins/day, 5 days/week, can be widely used in people's daily life, corresponding to about 7 hours of walking, 5 hours of aerobics, or 3 hours of lap swimming per week for men and 6 hours of walking, 4.5 hours of aerobics, or 2.5 hours of lap swimming per week for women [44, 45]. Compared with light physical activity, moderate to vigorous physical activity (exercise) has a more effective protection mechanism in PD patients. The moderate treadmill exercise and one-time exhaustion exercise, in addition to effectively reducing the risk of PD [1], can also enhance motor deficits in PD patients [54], improve cognitive impairment, and depression [50], which is consistent with the role of light physical activity. Besides, animal experiments demonstrated that moderate to vigorous physical exercise could enhance mitochondrial function [69] and reduce oxidative stress [70, 71] in the brain of PD mice. More importantly, exercise can reduce the accumulation of the pathogenic protein *α*-Syn and prevent neuronal apoptosis [71].

## 4. Molecular Mechanisms of Physical Exercise Relieving Parkinson's Disease

### 4.1. Physical Exercise Can Reduce the Accumulation of the *α*-Syn Protein

The *α*-syn protein is the main pathogenic protein of PD, which is acidic synaptophysin expressed in the vertebrate presynaptic. In the central nervous system (CNS), many neurodegenerative diseases are associated with the exiting of the *α*-syn protein in the cytoplasm and nucleus [72]. Aggregation of *α*-syn is a crucial risk factor for PD, multiple system atrophy (MSA), and Lewy body dementia (DLB) [73, 74]. In previous studies, physical exercise was found to have a positive effect on neurodegenerative diseases such as Huntington's disease, Alzheimer's disease, and PD [75–77]. Physical exercise could reduce the loss of dopaminergic neurons, increase synaptic connections, and upregulate neurotrophic factor levels to improve PD dyskinesia [78–80]. Physical exercise could downregulate *α*-Syn protein levels and neuronal apoptosis [70, 81–83], which could reduce inflammation and mitochondrial dysfunction to restore the motor function in PD patients. Overexpression of *α*-synuclein also resulted in significant impairment on hippocampal neurogenesis-dependent pattern separation (a cognitive task). Voluntary running exercise could prevent deterioration and improve cognition through the decrease of *α*-Syn protein overexpression. This can be further substantiated by an effect of running on neurogenesis levels in the dorsal dentate gyrus, suggesting that the functional effects of running on pattern separation were mediated via increased neurogenesis [84]. However, the exact molecular mechanisms of exercise-induced *α*-syn protein level decrease are unrevealed.

The accumulation of the *α*-Syn protein is the main reason for neuron loss [85, 86]. Physical exercise can significantly reduce *α*-Syn protein neuron loss in PD rodent models [70, 82, 83], but there are still inconsistent results from other studies [87, 88]. The different extent to the loss of neurons may be due to changes in exercise duration and intensity, which could affect the motor benefits of PD patients [89]. Aguiar et al. found that six weeks of running did not prevent MPTP neurotoxicity, suggesting that the duration of physical exercise should be prolonged to induce a neuroprotection effect [90]. Therefore, short-term, low-intensity physical exercise is not sufficient to alleviate neuronal loss [88], but moderate to intense intensity of physical exercise may have the protective effect on neurons from loss [70, 91, 92].

Active physical exercise can alleviate neuron loss and enhance nerve regeneration. In the substantia nigra pars compacta and striatum brain regions, exercise increases the levels of tyrosine hydroxylase (TH) and dopamine transporter (DAT) [82, 93–96], which will promote the expression of PSD-95 and synaptophysin [78, 97, 98]. These positive effects also could increase the function of dendritic spines on dopaminergic neurons and nerve fibers [99–102]. Current studies have found a significant reduction in neurogenesis in the hippocampus of PD patient, while a significant increase in neurogenesis after a period of running. Increased neurogenesis in the hippocampus promoted learning ability and memory function [103–105]. Both acute and chronic physical exercises could increase hippocampus activity [106, 107]. Exercise improves hippocampal synaptic plasticity mainly due to enhanced synaptic efficacy and expression of molecules involved in learning and memory [108–110]. Physical exercise not only promotes cell proliferation but also promotes the differentiation of newly formed nerve cells. Physical exercise increases neurogenesis, enhances synaptic plasticity in neurons, and improves spatial memory.

### 4.2. Physical Exercise Can Reduce Inflammation and Oxidative Stress

PD is a very common neurodegenerative disease in the elderly that is characterized by skeletal muscle abnormalities [111, 112]. Related studies reported that the inflammatory factors were upregulated in PD brain [113–115]. In addition, overexpression of inflammatory factors has also been shown in gastrocnemius skeletal muscle in PD, which suggested that they may play a role in the progression of skeletal muscle abnormalities [116]. Physical exercise training can reduce the risk of inflammation [117–126]. Erekat et al. and Al-Jarrah et al. found PD-induced changes in skeletal muscle IL-1*β* and TNF-*α* inflammatory cytokine expression. This is consistent with previous studies that skeletal muscle fibers are capable of producing proinflammatory factors [118, 127]. These proinflammatory cytokines are overexpressed in PD-induced skeletal muscle cells [116, 128, 129]. However, after physical training, overexpressed inflammatory cytokines are inhibited in aging skeletal muscle [64, 122]. Previous reports suggest that physical training promotes the expression of antioxidant enzymes, which may help reduce the production of proinflammatory cytokines in the skeletal muscle. Regular physical exercise can upregulate cellular antioxidant capacity and reduce the production rate of reactive oxygen species (ROS) [65–67, 130]. Besides, physical exercise can increase the number and function of mitochondria to induce mitochondrial biogenesis [68, 131–135], thereby improving the oxidative environment caused by mitochondrial abnormalities, which are associated with inflammatory reactions occurring in PD skeletal muscle [136]. Therefore, it can be assumed that mitochondrial biogenesis induced after physical exercise training leads to a decrease in proinflammatory factors in the skeletal muscle in PD patient [137, 138].

Accumulation of the *α*-syn protein in neurons leads to inflammation in the brain [139–141]. Aggregation of *α*-Syn induced the production of proinflammatory cytokines, which are toxic and thus cause cell death of dopaminergic neurons [142, 143]. Therefore, reducing the inflammatory response may be an effective way to deal with PD. Several studies found that physical exercise can improve the oxidative metabolism and the expression of antioxidant enzymes in the brain of mice [144–146]. Tuon et al. indicated that physical exercise was beneficial in reducing the production of proinflammatory proteins and inflammation in the brain of PD mice [91]. This result is consistent with previous studies by Sung et al. and Al-Jarrah et al., which suggested that physical exercise could decrease the level of proinflammatory proteins in the striatum and hippocampus in the PD experimental model through reducing the activity of microglia [147, 148].

Based on the above studies, we speculate that multiple pathways are involved in the regulation of physical exercise in PD animal experiments, such as alleviate the production of proinflammatory factors in the musculoskeletal muscle, reduce the expression of inflammatory factors in the brain, reduce the inflammatory response, and regulate oxidative stress [149, 150].

### 4.3. Physical Exercise Can Increase the Upregulation of Brain-Derived Neurotrophic Factor (BDNF)

The neuroprotective effects of exercise in PD may be promoted by neurotrophic factors, such as brain-derived neurotrophic factor (BDNF). BDNF plays a vital role in cell differentiation, neuronal survival, migration, synapse development, and synaptic plasticity [151]. Studies have shown that neurogenesis, combined with BDNF, can mimic the beneficial effects of physical exercise on mice [152]. Many studies have shown that physical exercise can produce antidepressant effects by increasing the level of BDNF, as well as prevent neurodegenerative diseases [66]. Running has been shown to increase BDNF and nerve growth factor (NGF) [153], fibroblast growth factor-2 (FGF-2) [154], and insulin-like growth factor 1 (IGF-1) [155]. Striatum trophic factors have potent trophic activity on DA neurons [156–159]. For example, 6-OHDA could prevent neurotoxic effects [160–162]. Physical exercise-trained rats showed an increase in protein and mRNA levels of trophic factors in the brain [153, 163], which suggested that this neuroprotection is caused by an increase in trophic factors [164, 165]. Besides, the protective effects induced by physical exercise are not limited to this aspect. We noted that FGF-2, a trophic factor, is upregulated after physical exercise [154] and has also been shown to induce expression of GDNF and BDNF in vitro [166, 167]. Physical exercise could increase the levels of BDNF and other neurotrophic factors, while toxins in the striatum and hippocampus could decrease those factors [168, 169]. In summary, studies have shown that physical exercise leads to the upregulation of neurotrophic factors in the brain of PD mice and enhances neuronal survival, differentiation, and synaptic plasticity in the central nervous system.

### 4.4. Physical Exercise Can Enhance Mitochondrial Function

Mitochondria are essential dynamic organelles responsible for the production of a large number of cellular energy molecules, adenosine triphosphate (ATP). Mitochondria also often undergo fission, fusion, and biogenesis and maintain tubular networks under normal conditions. These dynamic processes play an essential role in neuronal survival and homeostasis [170]. The common hallmark of several neurodegenerative diseases (Huntington's disease, Alzheimer's disease, and PD) is impaired function or expression of PGC-1*α*, a major regulator of mitochondrial biogenesis [171]. Recent studies have found that disrupting mitochondrial dynamics (e.g., excessive fission and repressed biosynthesis) leads to mitochondrial dysfunction in PD, which triggers neuronal cell death [172–174]. More and more researches have begun to reveal that endurance aerobic exercise can improve mitochondrial function in the brain [175]. Cells exposed to neurotoxins showed mitochondrial rupture, reduced mitochondrial protein, and cell death. Aerobic exercise could change mitochondrial phenotype, such as upregulating antiapoptotic protein (MCL-1 and BLC-2) and reducing proapoptotic proteins [70, 176]. Also, physical exercise could regulate fusion (such as MFN1/2 and OPA1) and fission [177–180] and enhance mitochondrial biosynthesis to promote mitochondrial dynamics [181]. The number of autophagic vacuoles increased in neurons in the brain regions of PD patients. This result indicates that the autophagy of neurons in the brains of PD patients is higher than that of the healthy population [182]. Koo et al. found that running exercise promotes autophagic clearance of mitochondrial *α*-Syn by activating mitochondrial silencing signal regulator-1 (SIRT1) [77]. Jang et al. suggested that eight weeks of running exercise regulates levels of autophagy-associated proteins, including microtubule-associated protein 1 light chain 3-II, p62, BECLIN1, BNIP3, and lysosomal-associated membrane protein-2. Those factors were all downregulated in the PD mice group while reversed in the exercise group. Therefore, physical exercise relieve PD symptoms in multiple ways, such as the autophagic ability promotion of the cells [183] and mitochondrial function enhancement.

## 5. Conclusion

The process of PD is affected by various factors, including risk factors and protective factors. As a safe treatment, physical activity could relieve the symptoms in PD patients, such as motor dysfunction, cognitive deficits, and depression. Different forms of physical activity, especially the moderate to vigorous physical exercise, have a positive impact on PD through multiple mechanisms, including reducing the accumulation of the *α*-syn protein and alleviating inflammation and oxidative stress, while enhancing BDNF activity, nerve regeneration, and mitochondrial function. So, there is an urgent need for illustrating the molecular mechanism of physical activity-induced protective effect on PD in order to provide the theoretical basis for improving the efficacy of prevention and treatment of PD (see Figure 1).

## References

[1] Ascherio A., Schwarzschild M. A. (2016). The epidemiology of Parkinson's disease: risk factors and prevention. *The Lancet Neurology*.

[2] Remy P., Doder M., Lees A., Turjanski N., Brooks D. (2005). Depression in Parkinson's disease: loss of dopamine and noradrenaline innervation in the limbic system. *Brain*.

[3] Xie A., Gao J., Xu L., Meng D. (2014). Shared Mechanisms of Neurodegeneration in Alzheimer’s Disease and Parkinson’s Disease. *BioMed Research International*.

[4] Crowley E. K., Nolan Y. M., Sullivan A. M. (2019). Exercise as a therapeutic intervention for motor and non-motor symptoms in Parkinson's disease: Evidence from rodent models. *Progress in Neurobiology*.

[5] Liu Z., Li T., Li P. (2015). The Ambiguous Relationship of Oxidative Stress, Tau Hyperphosphorylation, and Autophagy Dysfunction in Alzheimer’s Disease. *Oxidative Medicine and Cellular Longevity*.

[6] Lees A. J. (2012). The relevance of the Lewy body to the pathogenesis of idiopathic Parkinson's disease. *J Neurol Neurosurg Psychiatry*.

[7] Dawson T. M., Dawson V. L. (2010). The role of parkin in familial and sporadic Parkinson's disease. *Movement Disorders*.

[8] Lesage S., Brice A. (2012). Role of mendelian genes in "sporadic" Parkinson's disease. *Parkinsonism & Related Disorders*.

[9] Hauser D. N., Hastings T. G. (2013). Mitochondrial dysfunction and oxidative stress in Parkinson's disease and monogenic parkinsonism. *Neurobiology of Disease*.

[10] Wirdefeldt K., Adami H. O., Cole P., Trichopoulos D., Mandel J. (2011). Epidemiology and etiology of Parkinson's disease: a review of the evidence. *European Journal of Epidemiology*.

[11] Elbaz A., Bower J. H., Maraganore D. M. (2002). Risk tables for parkinsonism and Parkinson's disease. *Journal of Clinical Epidemiology*.

[12] Winkler A. S., Tütüncü E., Trendafilova A. (2010). Parkinsonism in a population of northern Tanzania: a community-based door-to-door study in combination with a prospective hospital-based evaluation. *Journal of Neurology*.

[13] Dotchin C., Msuya O., Kissima J. (2008). The prevalence of Parkinson's disease in rural Tanzania. *Movement Disorders*.

[14] Zhang Z., Roman G., Hong Z. (2005). Parkinson's disease in China: prevalence in Beijing, Xian, and Shanghai. *The Lancet*.

[15] Mayeux R., Marder K., Cote L. J. (1995). The frequency of idiopathic Parkinson's disease by age, ethnic group, and sex in northern Manhattan, 1988-1993. *American Journal of Epidemiology*.

[16] Van Den Eeden S. K., Tanner C. M., Bernstein A. L. (2003). Incidence of Parkinson's disease: variation by age, gender, and race/ethnicity. *American Journal of Epidemiology*.

[17] Wright Willis A., Evanoff B. A., Lian M., Criswell S. R., Racette B. A. (2010). Geographic and ethnic variation in Parkinson disease: a population-based study of US Medicare beneficiaries. *Neuroepidemiology*.

[18] Chen H., Zhang S. M., Hernán M. A., Willett W. C., Ascherio A. (2002). Diet and Parkinson's disease: a potential role of dairy products in men. *Annals of Neurology*.

[19] Chen H., O'Reilly E., McCullough M. L. (2007). Consumption of dairy products and risk of Parkinson's disease. *American Journal of Epidemiology*.

[20] Park M., Ross G. W., Petrovitch H. (2005). Consumption of milk and calcium in midlife and the future risk of Parkinson disease. *Neurology*.

[21] Petrovitch H., Ross G. W., Abbott R. D. (2002). Plantation work and risk of Parkinson disease in a population-based longitudinal study. *Archives of Neurology*.

[22] Ascherio A., Chen H., Weisskopf M. G. (2006). Pesticide exposure and risk for Parkinson's disease. *Annals of Neurology*.

[23] Tanner C. M., Kamel F., Ross G. W. (2011). Rotenone, paraquat, and Parkinson's disease. *Environmental Health Perspectives*.

[24] Marras C., Hincapié C. A., Kristman V. L. (2014). Systematic review of the risk of Parkinson's disease after mild traumatic brain injury: results of the International Collaboration on Mild Traumatic Brain Injury Prognosis. *Archives of Physical Medicine and Rehabilitation*.

[25] Hu G., Jousilahti P., Nissinen A., Antikainen R., Kivipelto M., Tuomilehto J. (2006). Body mass index and the risk of Parkinson disease. *Neurology*.

[26] Roos E., Grotta A., Yang F. (2018). Body mass index, sitting time, and risk of Parkinson disease. *Neurology*.

[27] Zhang D., Jiang H., Xie J. (2014). Alcohol intake and risk of Parkinson's disease: a meta-analysis of observational studies. *Movement Disorders*.

[28] Yamamoto T., Moriwaki Y., Takahashi S. (2005). Effect of ethanol on metabolism of purine bases (hypoxanthine, xanthine, and uric acid). *Clinica Chimica Acta*.

[29] Ross G. W., Abbott R. D., Petrovitch H. (2000). Association of coffee and caffeine intake with the risk of Parkinson disease. *JAMA*.

[30] Liu R., Guo X., Park Y. (2012). Caffeine intake, smoking, and risk of Parkinson disease in men and women. *American Journal of Epidemiology*.

[31] Ascherio A., Zhang S. M., Hernán M. A. (2001). Prospective study of caffeine consumption and risk of Parkinson's disease in men and women. *Annals of Neurology*.

[32] Hu G., Bidel S., Jousilahti P., Antikainen R., Tuomilehto J. (2007). Coffee and tea consumption and the risk of Parkinson's disease. *Movement Disorders*.

[33] Morens D. M., Grandinetti A., Reed D., White L. R., Ross G. W. (1995). Cigarette smoking and protection from Parkinson's disease: false association or etiologic clue?. *Neurology*.

[34] Hernán M. A., Zhang S. M., Rueda-DeCastro A. M., Colditz G. A., Speizer F. E., Ascherio A. (2001). Cigarette smoking and the incidence of Parkinson's disease in two prospective studies. *Annals of Neurology*.

[35] Chen H., Huang X., Guo X. (2010). Smoking duration, intensity, and risk of Parkinson disease. *Neurology*.

[36] Becker C., Jick S. S., Meier C. R. (2008). Use of antihypertensives and the risk of Parkinson disease. *Neurology*.

[37] Pasternak B., Svanström H., Nielsen N. M., Fugger L., Melbye M., Hviid A. (2012). Use of calcium channel blockers and Parkinson's disease. *American Journal of Epidemiology*.

[38] Gao X., Chen H., Fung T. T. (2007). Prospective study of dietary pattern and risk of Parkinson disease. *The American Journal of Clinical Nutrition*.

[39] Chen H., Zhang S. M., Schwarzschild M. A., Hernan M. A., Ascherio A. (2005). Physical activity and the risk of Parkinson disease. *Neurology*.

[40] Logroscino G., Sesso H. D., Paffenbarger R. S., Lee I. M. (2006). Physical activity and risk of Parkinson's disease: a prospective cohort study. *Journal of Neurology, Neurosurgery & Psychiatry*.

[41] Nelson L. M. (2018). Physical activity and Parkinson disease Risk. *JAMA Network Open*.

[42] Alwardat M., Schirinzi T., di Lazzaro G. (2019). Association between physical activity and dementia's risk factors in patients with Parkinson's disease. *Journal of Neural Transmission*.

[43] Sasco A. J., Paffenbarger R. S., Gendre I., Wing A. L. (1992). The role of physical exercise in the occurrence of Parkinson's disease. *Archives of Neurology*.

[44] Thacker E. L., Chen H., Patel A. V. (2008). Recreational physical activity and risk of Parkinson's disease. *Movement Disorders*.

[45] Xu Q., Park Y., Huang X. (2010). Physical activities and future risk of Parkinson disease. *Neurology*.

[46] Yang F., Trolle Lagerros Y., Bellocco R. (2015). Physical activity and risk of Parkinson's disease in the Swedish National March Cohort. *Brain*.

[47] The Cochrane Collaboration, Mehrholz J., Friis R. (2010). Treadmill training for patients with Parkinson's disease. *Cochrane Database of Systematic Reviews*.

[48] Herman T., Giladi N., Hausdorff J. M. (2009). Treadmill training for the treatment of gait disturbances in people with Parkinson's disease: a mini-review. *Journal of Neural Transmission*.

[49] Aguiar L. P. C., da Rocha P. A., Morris M. (2016). Therapeutic dancing for Parkinson's disease. *International Journal of Gerontology*.

[50] Shanahan J., Morris M. E., Bhriain O. N., Saunders J., Clifford A. M. (2015). Dance for people with Parkinson disease: what is the evidence telling us?. *Archives of Physical Medicine and Rehabilitation*.

[51] Song R., Grabowska W., Park M. (2017). The impact of Tai Chi and Qigong mind-body exercises on motor and non-motor function and quality of life in Parkinson's disease: a systematic review and meta-analysis. *Parkinsonism & Related Disorders*.

[52] Yang Y., Li X. Y., Gong L., Zhu Y. L., Hao Y. L. (2014). Tai Chi for improvement of motor function, balance and gait in Parkinson's disease: a systematic review and meta-analysis. *PLoS One*.

[53] Zhou J., Yin T., Gao Q., Yang X. C. (2015). A Meta-Analysis on the Efficacy of Tai Chi in Patients with Parkinson’s Disease between 2008 and 2014. *Evidence-Based Complementary and Alternative Medicine*.

[54] Keus S. H. J., Bloem B. R., Hendriks E. J. M., Bredero-Cohen A. B., Munneke M., on behalf of the Practice Recommendations Development Group (2007). Evidence-based analysis of physical therapy in Parkinson's disease with recommendations for practice and research. *Movement Disorders*.

[55] Kanegusuku H., Silva-Batista C., Pecanha T. (2017). Effects of progressive resistance training on cardiovascular autonomic regulation in patients with Parkinson disease: a randomized controlled trial. *Archives of Physical Medicine and Rehabilitation*.

[56] Asahina M., Vichayanrat E., Low D. A., Iodice V., Mathias C. J. (2013). Autonomic dysfunction in parkinsonian disorders: assessment and pathophysiology. *Journal of Neurology, Neurosurgery & Psychiatry*.

[57] Silva-Batista C., de Brito L. C., Corcos D. M. (2017). Resistance training improves sleep quality in subjects with moderate Parkinson's disease. *Journal of Strength and Conditioning Research*.

[58] Wassom D. J., Lyons K. E., Pahwa R., Liu W. (2015). Qigong exercise may improve sleep quality and gait performance in Parkinson's disease: a pilot study. *International Journal of Neuroscience*.

[59] David F. J., Robichaud J. A., Leurgans S. E. (2015). Exercise improves cognition in Parkinson's disease: the PRET-PD randomized, clinical trial. *Movement Disorders*.

[60] McKee K. E., Hackney M. E. (2013). The effects of adapted tango on spatial cognition and disease severity in Parkinson's disease. *Journal of Motor Behavior*.

[61] Cruise K. E., Bucks R. S., Loftus A. M., Newton R. U., Pegoraro R., Thomas M. G. (2011). Exercise and Parkinson's: benefits for cognition and quality of life. *Acta Neurologica Scandinavica*.

[62] Reynolds G. O., Otto M. W., Ellis T. D., Cronin-Golomb A. (2016). The therapeutic potential of exercise to improve mood, cognition, and sleep in Parkinson's disease. *Movement Disorders*.

[63] Teixeira-Machado L., Araújo F. M., Cunha F. A., Menezes M., Menezes T., Melo DeSantana J. (2015). Feldenkrais method-based exercise improves quality of life in individuals with Parkinson’s disease: a controlled, randomized clinical trial. *Alternative Therapies in Health and Medicine*.

[64] Greiwe J. S., Cheng B., Rubin D. C., Yarasheski K. E., Semenkovich C. F. (2001). Resistance exercise decreases skeletal muscle tumor necrosis factor *α* in frail elderly humans. *The FASEB Journal*.

[65] Lambertucci R. H., Levada-Pires A. C., Rossoni L. V., Curi R., Pithon-Curi T. C. (2007). Effects of aerobic exercise training on antioxidant enzyme activities and mRNA levels in soleus muscle from young and aged rats. *Mechanisms of Ageing and Development*.

[66] Linke A., Adams V., Schulze P. C. (2005). Antioxidative effects of exercise training in patients with chronic heart failure: increase in radical scavenger enzyme activity in skeletal muscle. *Circulation Journal*.

[67] Schulze P. C., Gielen S., Schuler G., Hambrecht R. (2002). Chronic heart failure and skeletal muscle catabolism: effects of exercise training. *International Journal of Cardiology*.

[68] Vettor R., Valerio A., Ragni M. (2014). Exercise training boosts eNOS-dependent mitochondrial biogenesis in mouse heart: role in adaptation of glucose metabolism. *American Journal of Physiology-Endocrinology and Metabolism*.

[69] Koo J. H., Jang Y. C., Hwang D. J. (2017). Treadmill exercise produces neuroprotective effects in a murine model of Parkinson's disease by regulating the TLR2/MyD88/NF-*κ*B signaling pathway. *Neuroscience*.

[70] Jang Y., Koo J. H., Kwon I. (2017). Neuroprotective effects of endurance exercise against neuroinflammation in MPTP-induced Parkinson's disease mice. *Brain Research*.

[71] Koo J. H., Cho J. Y. (2017). Erratum to: treadmill exercise attenuates *α*-Synuclein levels by promoting mitochondrial function and autophagy possibly via SIRT1 in the chronic MPTP/P-induced mouse model of Parkinson's disease. *Neurotoxicity Research*.

[72] Perrett S., Yu S., Chan P. (2014). Role of *α*-synuclein in neurodegeneration: implications for the pathogenesis of Parkinson's disease. *Essays in Biochemistry*.

[73] Jones E. (2009). *Just how special is Turkey in Europe?*.

[74] Nakata Y., Yasuda T., Fukaya M. (2012). Accumulation of *α*-Synuclein Triggered by Presynaptic Dysfunction. *Journal of Neuroscience*.

[75] Lauzé M., Daneault J.-F., Duval C. (2016). The effects of physical activity in Parkinson's disease: a review. *Journal of Parkinson's Disease*.

[76] Frese S., Petersen J. A., Ligon-Auer M. (2017). Exercise effects in Huntington disease. *Journal of Neurology*.

[77] Koo J. H., Kang E. B., Oh Y. S., Yang D. S., Cho J. Y. (2017). Treadmill exercise decreases amyloid-*β* burden possibly via activation of SIRT-1 signaling in a mouse model of Alzheimer's disease. *Experimental Neurology*.

[78] Toy W. A., Petzinger G. M., Leyshon B. J. (2014). Treadmill exercise reverses dendritic spine loss in direct and indirect striatal medium spiny neurons in the 1-methyl-4-phenyl-1,2,3,6-tetrahydropyridine (MPTP) mouse model of Parkinson's disease. *Neurobiology of Disease*.

[79] Wang Z., Guo Y., Myers K. G., Heintz R., Holschneider D. P. (2015). Recruitment of the prefrontal cortex and cerebellum in Parkinsonian rats following skilled aerobic exercise. *Neurobiology of Disease*.

[80] Tuon T., Valvassori S. S., Dal Pont G. C. (2014). Physical training prevents depressive symptoms and a decrease in brain-derived neurotrophic factor in Parkinson's disease. *Brain Research Bulletin*.

[81] Dimatelis J. J., Hendricks S., Hsieh J. (2013). Exercise partly reverses the effect of maternal separation on hippocampal proteins in 6-hydroxydopamine-lesioned rat brain. *Experimental Physiology*.

[82] Tuon T., Valvassori S. S., LOPES-BORGES J. (2012). Physical training exerts neuroprotective effects in the regulation of neurochemical factors in an animal model of Parkinson's disease. *Neuroscience*.

[83] Koo J. H., Cho J. Y., Lee U. B. (2017). Treadmill exercise alleviates motor deficits and improves mitochondrial import machinery in an MPTP-induced mouse model of Parkinson's disease. *Experimental Gerontology*.

[84] Crowley E. K., Nolan Y. M., Sullivan A. M. (2018). Neuroprotective effects of voluntary running on cognitive dysfunction in an *α*-synuclein rat model of Parkinson's disease. *Neurobiology of Aging*.

[85] Furukawa Y., Kish S. J., Fahn S. (2004). Dopa-responsive dystonia due to mild tyrosine hydroxylase deficiency. *Annals of Neurology*.

[86] Zhu Y., Zhang J., Zeng Y. (2012). Overview of tyrosine hydroxylase in Parkinson's disease. *CNS & Neurological Disorders - Drug Targets*.

[87] Hood R. L., Liguore W. A., Moore C., Pflibsen L., Meshul C. K. (2016). Exercise intervention increases spontaneous locomotion but fails to attenuate dopaminergic system loss in a progressive MPTP model in aged mice. *Brain Research*.

[88] Sconce M., Churchill M., Greene R., Meshul C. (2015). Intervention with exercise restores motor deficits but not nigrostriatal loss in a progressive MPTP mouse model of Parkinson's disease. *Neuroscience*.

[89] Shulman L. M., Katzel L. I., Ivey F. M. (2013). Randomized clinical trial of 3 types of physical exercise for patients with Parkinson disease. *JAMA Neurology*.

[90] Aguiar A. S., Tristão F. S. M., Amar M. (2014). Six weeks of voluntary exercise don't protect C57BL/6 mice against neurotoxicity of MPTP and MPP(+). *Neurotoxicity Research*.

[91] Tuon T., Souza P. S., Santos M. F. (2015). Physical Training Regulates Mitochondrial Parameters and Neuroinflammatory Mechanisms in an Experimental Model of Parkinson’s Disease. *Oxidative Medicine and Cellular Longevity*.

[92] Aguiar A. S., Lopes S. C., Tristão F. S. M. (2016). Exercise improves cognitive impairment and dopamine metabolism in MPTP-treated mice. *Neurotoxicity Research*.

[93] Hong Z., Wang G., Gu J. (2007). Tripchlorolide protects against MPTP-induced neurotoxicity in C57BL/6 mice. *European Journal of Neuroscience*.

[94] Bergen J. L., Toole T., Elliott III R. G., Wallace B., Robinson K., Maitland C. G. (2002). Aerobic exercise intervention improves aerobic capacity and movement initiation in Parkinson's disease patients. *NeuroRehabilitation*.

[95] Chen J., Qin J., Su Q., Liu Z., Yang J. (2012). Treadmill rehabilitation treatment enhanced BDNF-TrkB but not NGF-TrkA signaling in a mouse intracerebral hemorrhage model. *Neuroscience Letters*.

[96] Sim Y. J., Kim S. S., Kim J. Y., Shin M. S., Kim C. J. (2004). Treadmill exercise improves short-term memory by suppressing ischemia-induced apoptosis of neuronal cells in gerbils. *Neuroscience Letters*.

[97] Villalba R. M., Smith Y. (2010). Striatal spine plasticity in Parkinson's disease. *Frontiers in Neuroanatomy*.

[98] Hu S., Ying Z., Gomez-Pinilla F., Frautschy S. A. (2009). Exercise can increase small heat shock proteins (sHSP) and pre- and post- synaptic proteins in the hippocampus. *Brain Research*.

[99] Stranahan A. M., Khalil D., Gould E. (2007). Running induces widespread structural alterations in the hippocampus and entorhinal cortex. *Hippocampus*.

[100] Ribeiro R. P., Moreira E. L. G., Santos D. B. (2013). Probucol affords neuroprotection in a 6-OHDA mouse model of Parkinson's disease. *Neurochemical Research*.

[101] Ferreira A. F. B., Real C. C., Rodrigues A. C., Alves A. S., Britto L. R. G. (2010). Moderate exercise changes synaptic and cytoskeletal proteins in motor regions of the rat brain. *Brain Research*.

[102] El-Husseini A. E. D., Schnell E., Chetkovich D. M., Nicoll R. A., Bredt D. S. (2000). PSD-95 involvement in maturation of excitatory synapses. *Science*.

[103] Baek S. S., Jun T. W., Kim K. J., Shin M. S., Kang S. Y., Kim C. J. (2012). Effects of postnatal treadmill exercise on apoptotic neuronal cell death and cell proliferation of maternal-separated rat pups. *Brain and Development*.

[104] Duman R. S. (2005). Neurotrophic factors and regulation of mood: role of exercise, diet and metabolism. *Neurobiology of Aging*.

[105] van Praag H., Christie B. R., Sejnowski T. J., Gage F. H. (1999). Running enhances neurogenesis, learning, and long-term potentiation in mice. *Proceedings of the National Academy of Sciences*.

[106] Holschneider D. P., Maarek J. M. I., Yang J., Harimoto J., Scremin O. U. (2003). Functional brain mapping in freely moving rats during treadmill walking. *Journal of Cerebral Blood Flow & Metabolism*.

[107] Holschneider D. P., Yang J., Guo Y., Maarek J.-M. I. (2007). Reorganization of functional brain maps after exercise training: importance of cerebellar-thalamic-cortical pathway. *Brain Research*.

[108] Farmer J., Zhao X., van Praag H., Wodtke K., Gage F. H., Christie B. R. (2004). Effects of voluntary exercise on synaptic plasticity and gene expression in the dentate gyrus of adult male sprague-dawley rats _in vivo_. *Neuroscience*.

[109] Vaynman S., Ying Z., Gomez-Pinilla F. (2003). Interplay between brain-derived neurotrophic factor and signal transduction modulators in the regulation of the effects of exercise on synaptic-plasticity. *Neuroscience*.

[110] Vaynman S., Ying Z., Gomez-Pinilla F. (2004). Hippocampal BDNF mediates the efficacy of exercise on synaptic plasticity and cognition. *European Journal of Neuroscience*.

[111] Parkinson J. (2002). An essay on the shaking palsy. *The Journal of Neuropsychiatry and Clinical Neurosciences*.

[112] Reeve A., Simcox E., Turnbull D. (2014). Ageing and Parkinson's disease: why is advancing age the biggest risk factor?. *Ageing research reviews*.

[113] Kempuraj D., Thangavel R., Natteru P. A. (2016). Neuroinflammation induces neurodegeneration. *Journal of Neurology, Neurosurgery and Spine*.

[114] Li M., Dai F. R., du X. P., Yang Q. D., Chen Y. (2012). Neuroprotection by silencing iNOS expression in a 6-OHDA model of Parkinson's disease. *Journal of Molecular Neuroscience*.

[115] Lofrumento D. D., Saponaro C., Cianciulli A. (2011). MPTP-induced neuroinflammation increases the expression of pro-inflammatory cytokines and their receptors in mouse brain. *Neuroimmunomodulation*.

[116] Erekat N. S., Al-Jarrah M. D. (2018). Interleukin-1 beta and tumor necrosis factor alpha upregulation and nuclear factor kappa B activation in skeletal muscle from a mouse model of chronic/progressive Parkinson disease. *Medical science monitor: international medical journal of experimental and clinical research*.

[117] Briones A. M., Touyz R. M. (2009). Moderate exercise decreases inflammation and oxidative stress in hypertension: but what are the mechanisms?. *Hypertension*.

[118] Gielen S., Adams V., Möbius-Winkler S. (2003). Anti-inflammatory effects of exercise training in the skeletal muscle of patients with chronic heart failure. *Journal of the American College of Cardiology*.

[119] Gleeson M., Bishop N. C., Stensel D. J., Lindley M. R., Mastana S. S., Nimmo M. A. (2011). The anti-inflammatory effects of exercise: mechanisms and implications for the prevention and treatment of disease. *Nature Reviews Immunology*.

[120] Kadoglou N. P., Perrea D., Iliadis F., Angelopoulou N., Liapis C., Alevizos M. (2007). Exercise reduces resistin and inflammatory cytokines in patients with type 2 diabetes. *Diabetes Care*.

[121] Munk P. S., Breland U. M., Aukrust P., Ueland T., Kvaløy J. T., Larsen A. I. (2011). High intensity interval training reduces systemic inflammation in post-PCI patients. *European Journal of Cardiovascular Prevention & Rehabilitation*.

[122] Nicklas B. J., Brinkley T. E. (2009). Exercise training as a treatment for chronic inflammation in the elderly. *Exercise and Sport Sciences Reviews*.

[123] Packer N., Hoffman-Goetz L. (2012). Exercise training reduces inflammatory mediators in the intestinal tract of healthy older adult mice. *Canadian Journal on Aging / La Revue canadienne du vieillissement*.

[124] Phillips M. D., Patrizi R. M., Cheek D. J., Wooten J. S., Barbee J. J., Mitchell J. B. (2012). Resistance training reduces subclinical inflammation in obese, postmenopausal women. *Medicine & Science in Sports & Exercise*.

[125] Teixeira-Lemos E., Nunes S., Teixeira F., Reis F. (2011). Regular physical exercise training assists in preventing type 2 diabetes development: focus on its antioxidant and anti-inflammatory properties. *Cardiovasc Diabetol*.

[126] Zoppini G., Targher G., Zamboni C. (2006). Effects of moderate-intensity exercise training on plasma biomarkers of inflammation and endothelial dysfunction in older patients with type 2 diabetes. *Nutrition, Metabolism and Cardiovascular Diseases*.

[127] Saghizadeh M., Ong J. M., Garvey W. T., Henry R. R., Kern P. A. (1996). The expression of TNF alpha by human muscle. Relationship to insulin resistance. *Journal of Clinical Investigation*.

[128] Erekat N. S. (2015). Apoptotic mediators are upregulated in the skeletal muscle of chronic/progressive mouse model of Parkinson's disease. *The Anatomical Record*.

[129] Erekat N. S. (2017). Cerebellar Purkinje cells die by apoptosis in the shaker mutant rat. *Brain Research*.

[130] Samjoo I. A., Safdar A., Hamadeh M. J., Raha S., Tarnopolsky M. A. (2013). The effect of endurance exercise on both skeletal muscle and systemic oxidative stress in previously sedentary obese men. *Nutrition & Diabetes*.

[131] Kim B., Lee H., Kawata K., Park J. Y. (2014). Exercise-mediated wall shear stress increases mitochondrial biogenesis in vascular endothelium. *PLoS One*.

[132] Ljubicic V., Joseph A. M., Saleem A. (2010). Transcriptional and post-transcriptional regulation of mitochondrial biogenesis in skeletal muscle: effects of exercise and aging. *Biochimica et Biophysica Acta (BBA)-General Subjects*.

[133] Margolis L. M., Pasiakos S. M. (2013). Optimizing intramuscular adaptations to aerobic exercise: effects of carbohydrate restriction and protein supplementation on mitochondrial biogenesis. *Advances in Nutrition*.

[134] Ojuka E. O., Jones T. E., Han D.-H. O., Chen M., Holloszy J. O. (2003). Raising Ca2+ in L6 myotubes mimics effects of exercise on mitochondrial biogenesis in muscle. *FASEB J [J].*.

[135] Palmer H. S. (2010). Exercise training for a time-poor generation: enhanced skeletal muscle mitochondrial biogenesis. *The Journal of Physiology*.

[136] Song Y., Miao Y., Song C. P. (2014). Behind the scenes: the roles of reactive oxygen species in guard cells. *New Phytologist*.

[137] Gdynia H.-J., Sperfeld A. D., Unrath A. (2009). Histopathological analysis of skeletal muscle in patients with Parkinson's disease and 'dropped head'/'bent spine' syndrome. *Parkinsonism & Related Disorders*.

[138] Wang P., du Y., Hou Y. J. (2015). Nitric oxide negatively regulates abscisic acid signaling in guard cells by S-nitrosylation of OST1. *Proceedings of the National Academy of Sciences*.

[139] Codolo G., Plotegher N., Pozzobon T. (2013). Triggering of inflammasome by aggregated *α*–Synuclein, an inflammatory response in synucleinopathies. *PLoS One*.

[140] Drouin-Ouellet J., Gibrat C., Bousquet M., Calon F., Kriz J., Cicchetti F. (2011). The role of the MYD88-dependent pathway in MPTP-induced brain dopaminergic degeneration. *Journal of Neuroinflammation*.

[141] Hayward J. H., Lee S. J. (2014). A decade of research on TLR2 discovering its pivotal role in glial activation and neuroinflammation in neurodegenerative diseases. *Experimental Neurobiology*.

[142] Eikelenboom P., Veerhuis R., Scheper W., Rozemuller A. J. M., van Gool W. A., Hoozemans J. J. M. (2006). The significance of neuroinflammation in understanding Alzheimer's disease. *Journal of Neural Transmission*.

[143] Hensley K., Abdel-Moaty H., Hunter J. (2006). Primary glia expressing the G93A-SOD1 mutation present a neuroinflammatory phenotype and provide a cellular system for studies of glial inflammation. *Journal of Neuroinflammation*.

[144] Aguiar A. S., Tuon T., Soares F. S., da Rocha L. G. C., Silveira P. C., Pinho R. A. (2008). The effect of n-acetylcysteine and deferoxamine on exercise-induced oxidative damage in striatum and hippocampus of mice. *Neurochemical Research*.

[145] Somani S. M., Ravi R., Rybak L. P. (1995). Effect of exercise training on antioxidant system in brain regions of rat. *Pharmacology Biochemistry and Behavior*.

[146] Tsou Y. H., Shih C. T., Ching C. H. (2015). Treadmill exercise activates Nrf2 antioxidant system to protect the nigrostriatal dopaminergic neurons from MPP^+^ toxicity. *Experimental Neurology*.

[147] Sung Y.-H., Kim S. C., Hong H. P. (2012). Treadmill exercise ameliorates dopaminergic neuronal loss through suppressing microglial activation in Parkinson's disease mice. *Life Sciences*.

[148] Al-Jarrah M. (2013). Endurance exercise training protects against the upregulation of nitric oxide in the striatum of MPTP/probenecid mouse model of Parkinson's disease. *NeuroRehabilitation*.

[149] Wang P., du Y., Zhao X., Miao Y., Song C. P. (2013). The MPK6-ERF6-ROS-responsive cis-acting Element7/GCC box complex modulates oxidative gene transcription and the oxidative response in Arabidopsis. *Plant Physiology*.

[150] Wang P., du Y., Li Y., Ren D., Song C. P. (2010). Hydrogen peroxide-mediated activation of MAP kinase 6 modulates nitric oxide biosynthesis and signal transduction inArabidopsis. *The Plant Cell*.

[151] Cotman C. W., Berchtold N. C. (2002). Exercise: a behavioral intervention to enhance brain health and plasticity. *Trends in Neurosciences*.

[152] Choi S. H., Bylykbashi E., Chatila Z. K. (2018). Combined adult neurogenesis and BDNF mimic exercise effects on cognition in an Alzheimer's mouse model. *Science*.

[153] Neeper S. A., Gómez-Pinilla F., Choi J., Cotman C. W. (1996). Physical activity increases mRNA for brain-derived neurotrophic factor and nerve growth factor in rat brain. *Brain Research*.

[154] Gomez-Pinilla F., Vu L., Cotman C. W. (1995). Regulation of astrocyte proliferation by FGF-2 and heparan sulfate in vivo. *The Journal of Neuroscience*.

[155] Carro E., Trejo J. L., Busiguina S., Torres-Aleman I. (2001). Circulating insulin-like growth factor I mediates the protective effects of physical exercise against brain insults of different etiology and anatomy. *The Journal of Neuroscience*.

[156] Hyman C., Hofer M., Barde Y. A. (1991). BDNF is a neurotrophic factor for dopaminergic neurons of the substantia nigra. *Nature*.

[157] Altar C. A., Boylan C. B., Jackson C. (1992). Brain-derived neurotrophic factor augments rotational behavior and nigrostriatal dopamine turnover in vivo. *Proceedings of the National Academy of Sciences*.

[158] Lin L. F., Doherty D., Lile J., Bektesh S., Collins F. (1993). GDNF: a glial cell line-derived neurotrophic factor for midbrain dopaminergic neurons. *Science*.

[159] Winkler C., Sauer H., Lee C. S., Björklund A. (1996). Short-term GDNF treatment provides long-term rescue of lesioned nigral dopaminergic neurons in a rat model of Parkinson's disease. *The Journal of Neuroscience*.

[160] Schatz D. S., Kaufmann W. A., Saria A., Humpel C. (1999). Dopamine neurons in a simple GDNF-treated meso-striatal organotypic co-culture model. *Experimental Brain Research*.

[161] Shults C. W., Ray J., Tsuboi K., Gage F. H. (2000). Fibroblast growth factor-2-producing fibroblasts protect the nigrostriatal dopaminergic system from 6-hydroxydopamine. *Brain Research*.

[162] Wang L., Muramatsu S., Lu Y. (2002). Delayed delivery of AAV-GDNF prevents nigral neurodegeneration and promotes functional recovery in a rat model of Parkinson's disease. *Gene Therapy*.

[163] Gómez-Pinilla F., So V., Kesslak J. P. (1998). Spatial learning and physical activity contribute to the induction of fibroblast growth factor: neural substrates for increased cognition associated with exercise. *Neuroscience*.

[164] Duan W., Guo Z., Mattson M. P. (2001). Brain-derived neurotrophic factor mediates an excitoprotective effect of dietary restriction in mice. *Journal of Neurochemistry*.

[165] Young D., Lawlor P. A., Leone P., Dragunow M., During M. J. (1999). Environmental enrichment inhibits spontaneous apoptosis, prevents seizures and is neuroprotective. *Nature Medicine*.

[166] Suter-Crazzolara C., Unsicker K. (1996). GDNF mRNA levels are induced by FGF-2 in rat C6 glioblastoma cells. *Molecular Brain Research*.

[167] Kwon Y. K. (1997). Expression of brain-derived neurotrophic factor mRNA stimulated by basic fibroblast growth factor and platelet-derived growth factor in rat hippocampal cell line. *Molecules and Cells*.

[168] Rangasamy S. B., Soderstrom K., Bakay R. A., Kordower J. H. (2010). Neurotrophic factor therapy for Parkinson’s disease. *Progress in brain research*.

[169] Marais L., Stein D. J., Daniels W. M. U. (2009). Exercise increases BDNF levels in the striatum and decreases depressive-like behavior in chronically stressed rats. *Metabolic Brain Disease*.

[170] Onyango I. G., Lu J., Rodova M., Lezi E., Crafter A. B., Swerdlow R. H. (2010). Regulation of neuron mitochondrial biogenesis and relevance to brain health. *Biochimica et Biophysica Acta (BBA)-Molecular Basis of Disease*.

[171] Valero T. (2014). Editorial (Thematic Issue: Mitochondrial biogenesis: pharmacological Approaches). *Current Pharmaceutical Design*.

[172] Anandhan A., Jacome M. S., Lei S. (2017). Metabolic dysfunction in Parkinson's disease: bioenergetics, redox homeostasis and central carbon metabolism. *Brain Research Bulletin*.

[173] Bueler H. (2009). Impaired mitochondrial dynamics and function in the pathogenesis of Parkinson's disease. *Experimental Neurology*.

[174] Alvarez-Mora M. I., Rodriguez-Revenga L., Madrigal I., Guitart-Mampel M., Garrabou G., Mila M. (2017). Impaired mitochondrial function and dynamics in the pathogenesis of FXTAS. *Molecular Neurobiology*.

[175] Lu Y., Dong Y., Tucker D. (2017). Treadmill exercise exerts Neuroprotection and regulates microglial polarization and oxidative stress in a streptozotocin-induced rat model of sporadic Alzheimer's disease. *Journal of Alzheimer's Disease*.

[176] Jang Y., Kwon I., Song W., Cosio-Lima L. M., Lee Y. (2018). Endurance exercise mediates neuroprotection against MPTP-mediated Parkinson's disease via enhanced neurogenesis, antioxidant capacity, and autophagy. *Neuroscience*.

[177] Campos J. C., Queliconi B. B., Bozi L. H. M. (2017). Exercise reestablishes autophagic flux and mitochondrial quality control in heart failure. *Autophagy*.

[178] Chen C. C. W., Erlich A. T., Hood D. A. (2018). Role of Parkin and endurance training on mitochondrial turnover in skeletal muscle. *Skelet Muscle*.

[179] Jiang H.-K., Wang Y. H., Sun L. (2014). Aerobic interval training attenuates mitochondrial dysfunction in rats post-myocardial infarction: roles of mitochondrial network dynamics. *International Journal of Molecular Sciences*.

[180] Konopka A. R., Suer M. K., Wolff C. A., Harber M. P. (2014). Markers of human skeletal muscle mitochondrial biogenesis and quality control: effects of age and aerobic exercise training. *The Journals of Gerontology: Series A*.

[181] Heden T. D., Ryan T. E., Ferrara P. J. (2017). Greater oxidative capacity in primary myotubes from endurance-trained women. *Medicine & Science in Sports & Exercise*.

[182] Southern W. M., Nichenko A. S., Shill D. D. (2017). Skeletal muscle metabolic adaptations to endurance exercise training are attainable in mice with simvastatin treatment. *PLoS One*.

[183] Jang Y. C., Hwang D. J., Koo J. H. (2018). Association of exercise-induced autophagy upregulation and apoptosis suppression with neuroprotection against pharmacologically induced Parkinson's disease. *Journal of Exercise Nutrition & Biochemistry*.

